# Efficacy of salt reduction for managing overactive bladder symptoms: a prospective study in patients with excessive daily salt intake

**DOI:** 10.1038/s41598-021-83725-9

**Published:** 2021-02-18

**Authors:** Tomohiro Matsuo, Yasuyoshi Miyata, Asato Otsubo, Yuta Mukae, Kensuke Mitsunari, Kojiro Ohba, Hideki Sakai

**Affiliations:** Department of Urology, Nagasaki University Graduate School of Biomedical Sciences, 1-7-1 Sakamoto, Nagasaki, 852-8501 Japan

**Keywords:** Health care, Urology

## Abstract

This study aimed to investigate the efficacy of salt intake restriction on overactive bladder (OAB) symptoms in patients with excessive salt intake. Patients received a brochure on nutritional guidance regarding salt intake reduction and received health education every 4 weeks for 12 weeks. Data from overactive bladder symptom score (OABSS) questionnaires and frequency volume charts (FVCs) were evaluated. The daily salt intake was estimated by determining the urinary sodium and creatinine concentrations using spot urine samples. Of the 98 patients included, 71 (72.4%) successfully restricted their daily salt intake after 12 weeks (salt restricted [R] group), while 27 (27.6%) did not (salt non-restricted [N-R] group). The scores to each OABSS question and the resulting total score improved significantly in the R group; however, the individual scores remained unchanged and the total score increased in the N-R group. The FVC data indicated improved voided volumes in the R group as compared to in the N-R group. Ultimately, 17 (23.9%) patients in the R group no longer fulfilled the OAB diagnostic criteria after salt intake reduction. Thus, salt intake reduction improved urinary symptoms in patients with OAB and may be a therapeutic option for OAB in patients with excessive daily salt intakes.

## Introduction

Overactive bladder (OAB) is a symptom-based syndrome that is characterized by urinary urgency with or without urge incontinence^1^. Individuals with OAB experience urgency and other lower urinary tract symptoms (LUTS). Data from some epidemiological surveys suggest that the prevalence of OAB is between 16 and 19%^2–4^. In clinical practice, anticholinergic agents and/or β3 adrenergic agonists are often prescribed to patients with OAB; however, adverse events may occur due to the deterioration of physiological function, especially in the elderly, and administering additional oral medications would be difficult.

Poor eating habits can cause lifestyle-related diseases and the metabolic syndrome (MetS), which can contribute to the onset of OAB^5^. In addition, a routinely excessive salt intake is associated with MetS and lifestyle-related diseases (including hypertension, chronic kidney dysfunction, and diabetes mellitus), and induces symptoms associated with OAB^6,7^. Hence, improving the dietary habits might be an effective treatment option for OAB. In fact, some researchers have reported a relationship between diet and OAB^8,9^; however, little is known about the mechanism of OAB control following dietary improvements^10,11^.

We previously reported that reducing the salt intake improved nocturia and nocturnal polyuria^12^. Consequently, we have focused on the dietary modifications that may improve urinary disorders. Basic research using several animal models has shown that an excessive salt intake enhances the expression of oxidative stress genes and neurotransmitters and induces bladder ischemia, which causes OAB^13,14^. Furthermore, salt reduction has been shown to improve LUTS and these risk factors^13,14^. However, the effect of salt reduction on OAB in humans is not well understood. We hypothesized that salt intake reduction is a useful approach for managing OAB. Hence, the aim of this study was to examine the efficacy of salt intake restriction for the management of OAB in patients with excessive salt intakes.

## Methods

### Patients and nutritional guidance

This study was approved by our institution’s ethical committee. All participants who lived in Nagasaki city, Nagasaki, Japan provided a written informed consent. This prospective clinical study was performed from September 2014 to March 2015. Patients were included if they: (1) had symptoms of OAB for at least 3 months (overactive bladder symptom score [OABSS] ≥ 2 for urgency and a total OABSS ≥ 3^15^), (2) were either not taking any medications for OAB or were not responding satisfactorily to β3 adrenergic receptor agonists and/or anticholinergic agents for at least 3 months (OABSS: urgency ≥ 2, total score ≥ 3), and (3) had excessive salt intake, i.e., ≥ 8 g/day for men and 7 g/day for women^16^. Patients were excluded if they: (1) had acute urinary tract infections, (2) had any condition that affected the urinary function (including a history of pelvic surgery, radiation therapy, urethral strictures, pelvic organ prolapse, urological malignancies, or a neurogenic bladder), (3) were administered medications that could affect sodium (Na) excretion (for example, diuretics and antidiuretic hormone), or (4) had end-stage renal disease (which was defined as an estimated glomerular filtration rate [eGFR] < 15 mL/min/1.73 m^2^). Medications for OAB, such as β3 adrenergic receptor agonists and anticholinergic agents, were continued on the condition that the dose was kept constant during the study period.

The enrolled patients were given a brochure that provided nutritional guidance on salt intake restrictions. The brochure detailed the approximate salt intake from a regular diet and also described the use of salt for seasoning food. Furthermore, the brochure also described how to season food using spices, instead of salt, to limit the salt intake. The study spanned 4 weeks, and the patients received health education from the physicians and nurses every 4 weeks during the study.

### Urinary parameters and daily salt intake

To estimate the participants’ daily salt intake, we used morning spot urine samples that comprised of one sample per participant and were provided once a day between 9:00 am and 11:00 am at our institution, as described previously^17^. Thereafter, to estimate the Na and creatinine (Cr) concentrations in the spot urine samples from the daily salt intake, we used a formula that was adjusted for body height, weight, and age as follows: 24-h Na excretion (mEq/day) = 21.98 (Na S/[Cr S × 10] × Pr.UCr24)^0.392^ − 2.04 × age (years) + 14.89 × body weight (kg) + 16.14 × height (cm) − 2244.45, where Na S is the Na concentration in the spot urine sample in mEq/L, Cr S is the Cr concentration in the spot urine sample in mg/dL, and Pr.UCr24 is the predicted 24-h urinary Cr excretion in mg/day. This formula appeared to be less affected by the sample collection time, and its accuracy was relatively high.

On the basis of the International Continence Society’s standard, the nocturnal urine volume (NUV) was defined as the total volume of urine passed during the night, including the first morning void. Nocturnal polyuria index (NPi) was defined as the ratio of the nocturnal urine production to the 24-h total urine production. Na concentration in the spot urine test was represented as Na concentration in the spot voiding urine. Renal dysfunction was defined as an eGFR < 60 mL/min/1.73 m^2^.

We evaluated the objective and subjective urological parameters before and 12 weeks after salt intake reduction. We also evaluated the changes in the OAB using the OABSS questionnaire and assessed the patients’ salt intake by using spot urine samples to estimate their urinary Na excretion. In addition, we asked the participants to complete 3-day frequency volume charts (FVCs), and evaluated the average urinary volumes and voiding frequencies from these data. We defined successful salt reduction as a restriction in the absolute value of the estimated salt intake from baseline to 12 weeks after study initiation. The primary outcome in this study was the effectiveness of a reduction in the salt intake on the total OABSS scores in OAB patients who successfully reduced their salt intake. The secondary endpoints were the effects on each parameter in the OABSS list, objective findings in patients who successfully reduced their salt intake, and comparative subjective and objective findings between patients who reduced and did not reduce their salt intake successfully.

### Statistical analyses

The number of samples was determined on the basis of our previous report^12^. In our previous study, the success rate of salt reduction in the study subjects was 69.5%. Hence, we set a probability of 0.05 (two-sided), a power of 80%, and an effect size of 0.5. We estimated that the ideal number of participants for this study should be at least 75. All data are expressed as means ± standard deviations. The Student's *t*-test was used to compare the continuous parametric variables between patients with and without successful salt restriction. All statistical tests were performed using JMP, version 13 (SAS Institute Inc., Cary, NC, USA).

### Research involving human participants

This study was approved by the Nagasaki University Hospital Ethical Committee and carried out in accordance with the principles of the Declaration of Helsinki. All participants provided a written informed consent.

## Results

### Patients’ characteristics

A total of 98 patients (comprising of 52 men and 46 women) who fulfilled the criteria for OAB were enrolled in the study. As shown in Table 1, the mean age was 66.7 ± 11.5 years. Around 71 patients (72.4%) successfully reduced their salt intake and were included in the salt restricted (R) group, while 27 patients (27.6%) did not succeed in reducing their salt intake and were included in the salt non-restricted (N-R) group. There were no differences between the two groups in terms of the body mass index or the proportion of comorbidities such as hypertension, diabetes mellitus, and hyperlipidemia. The estimated mean salt intake at baseline was significantly higher in the R group (10.4 ± 2.2 g) than in the N-R group (9.6 ± 0.9 g) (*p* < 0.001). In this study, there were no missing values in the FVCs, and all items could be analyzed in all patients. Table 1 also shows the differences in the subjective and objective symptoms at baseline between the two groups. Furthermore, the groups did not differ in terms of the urological parameters determined from the 3-day FVCs and the data on subjective symptoms gathered from the OABSS questionnaires.

**Table 1:** Patients’ characteristics and urological symptoms before salt intake reduction in each group.

	Entire cohort (n = 98)	R group (n = 71)	N-R group (n = 27)	*p* value
Sex; male/female	52/46	34/37	18/9	0.116
Age (years); mean ± SD	66.7 ± 11.5	67.7 ± 11.2	64.1 ± 12.0	0.175
Body mass index (kg/m^2^)	22.7 ± 3.5	22.4 ± 3.7	23.6 ± 3.0	0.140
Duration of illness (months)	19.7 ± 17.1	23.5 ± 20.5	18.4 ± 15.8	0.217
**Drug for overactive bladder; n (%)**	64 (65.3)	48 (67.6)	16 (59.3)	0.321
β3-adrenoceptor agonists	48 (75.0)	38 (79.2)	10 (62.5)	
Antimuscarinic agents	16 (25.0)	10 (20.8)	6 (37.5)	
Hypertension (%)	50 (59.2)	41 (57.8)	17 (63.0)	0.818
Diabetes mellitus (%)	16 (16.3)	13 (18.3)	3 (11.0)	0.545
Renal dysfunction (%)	31 (31.6)	21 (29.6)	10 (37.0)	0.478
Hyperlipidemia (%)	14 (14.3)	10 (14.1)	4 (14.8)	1.000
Fluid intake volume (mL/day)	2536.7 ± 627.4	2448.7 ± 699.7	2463.3 ± 559.4	0.923
*SUNa (mEq/L)	105.1 ± 45.7	108.5 ± 44.0	96.1 ± 49.5	0.001
Est. 24-h U-Na excretion (mEq/day)	173.3 ± 34.5	176.7 ± 38.8	164.3 ± 16.0	< 0.001
Estimated daily salt intake (g/day)	10.2 ± 1.9	10.4 ± 2.2	9.6 ± 0.9	< 0.001
Daytime frequency	8.4 ± 2.8	8.4 ± 2.8	8.4 ± 2.8	0.923
Nighttime frequency	2.5 ± 0.9	2.5 ± 1.0	2.3 ± 0.9	0.284
Voided volume (mL)	238.1 ± 22.2	247.8 ± 25.1	240.9 ± 24.0	0.616
Diurnal urine volume (mL)	1790.8 ± 559.5	1784.5 ± 585.3	1807.4 ± 495.3	0.620
Nocturnal urine volume (mL)	745.9 ± 188.8	757.0 ± 193.7	716.7 ± 175.4	0.284
Nocturnal polyuria index (%)	30.2 ± 7.2	30.7 ± 7.6	29.0 ± 6.3	0.459
**OABSS**				
Q1. Daytime frequency	1.2 ± 1.1	1.2 ± 1.0	1.2 ± 1.4	0.993
Q2. Nocturia	2.1 ± 0.6	2.1 ± 0.5	2.1 ± 0.6	0.788
Q3. Urgency	2.3 ± 0.5	2.3 ± 0.5	2.2 ± 0.4	0.327
Q4. Urgency incontinence	1.3 ± 1.0	1.3 ± 1.0	1.3 ± 0.8	0.774
Total score	6.8 ± 1.9	6.9 ± 1.0	6.8 ± 2.0	0.775

*R group* restricted group, *N-R group* non-restricted group, *SUNa* Na concentration in the spot urine, *Est. 24-h U-Na* estimated 24-h urinary Na, *OABSS* overactive bladder symptom score, *QI, Q2, Q3, and Q4* questions 1, 2, 3, and 4, respectively.

### Changes in the salt intake volume and the urological parameters assessed using the FVC and OABSS

Table 2 shows the changes in the estimated salt intakes and in the urological parameters determined from the 3-day FVCs. During the study period, the patients in the R group reduced their mean daily salt intake from 10.4 ± 2.2 g/day to 7.7 ± 2.0 g/day (*p* < 0.001), while the patients in the N-R group increased their mean daily salt intake from 9.6 ± 0.9 g/day to 11.5 ± 1.9 g/day (*p* < 0.001). According to the 3-day FVC data, in the R group, the mean daytime and nighttime voiding frequencies decreased significantly after nutritional guidance (*p* < 0.001; Table 2). Conversely, in the N-R group, the mean daytime and nighttime voiding frequencies increased significantly, regardless of whether the patients received nutritional guidance or not (*p* = 0.015 and 0.043, respectively; Table 2). However, the fluid intake volume in the R group decreased from 2,448.7 ± 699.7 mL/day to 2,066.2 ± 608.0 mL/day (*p* < 0.001), while the volume in the N-R group increased significantly from 2,463.3 ± 559.4 mL/day to 2,735.2 ± 556.7 mL/day (*p* < 0.001). Furthermore, the voided volume in the R group increased from 247.8 ± 25.1 mL to 260.4 ± 32.6 mL (*p* < 0.001), while the voided volume in the N-R group decreased from 240.9 ± 24.0 mL to 238.3 ± 24.1 mL (*p* = 0.007). The diurnal urine volume, NUV, and NPi decreased significantly after nutritional guidance in the R group; however, a significant decrease was not found in the diurnal urine volume of the N-R group (Table 2). Furthermore, the changes in the least square mean of the NPi from the baseline to 12 weeks later were -8.5% in the R-group and -0.2% in the N-R group (P = 0.012).

**Table 2:** Changes in salt intake volume and urological parameters including overactive bladder symptom scorer between two groups.

	R group			N-R group		
	0 week	12 weeks	*p* value	0 week	12 weeks	*p* value
SUNa (mEq/L); mean ± SD	108.5 ± 44.0	87.2 ± 40.9	< 0.001	96.1 ± 49.5	121.1 ± 53.9	0.005
Est. 24-h U-Na excretion (mEq/day)	176.7 ± 38.8	132.4 ± 33.8	< 0.001	164.3 ± 16.0	196.4 ± 32.7	< 0.001
Estimated daily salt intake (g/day)	10.4 ± 2.2	7.7 ± 2.0	< 0.001	9.6 ± 0.9	11.5 ± 1.9	< 0.001
Daytime frequency	8.4 ± 2.8	6.8 ± 2.4	< 0.001	8.4 ± 2.8	9.0 ± 2.7	0.015
Nighttime frequency	2.5 ± 1.0	1.6 ± 0.9	< 0.001	2.3 ± 0.9	2.6 ± 1.0	0.043
Fluid intake volume (mL)	2448.7 ± 699.7	2066.2 ± 608.0	< 0.001	2463.3 ± 559.4	2735.2 ± 556.7	< 0.001
Voided volume (mL)	247.8 ± 25.1	260.4 ± 32.6	< 0.001	240.9 ± 24.0	238.3 ± 24.1	0.007
Diurnal UV (mL)	1784.5 ± 585.3	1461.7 ± 494.3	< 0.001	1807.4 ± 495.3	1946.3 ± 461.4	0.132
Nocturnal UV (mL)	757.0 ± 193.7	571.1 ± 186.6	< 0.001	716.7 ± 175.4	775.9 ± 201.1	0.043
Nocturnal polyuria index (%)	30.7 ± 7.6	28.6 ± 7.2	0.007	29.0 ± 6.3	28.9 ± 6.8	0.850
**OABSS**						
Q1. Diurnal frequency	1.2 ± 1.0	0.6 ± 1.0	< 0.001	1.2 ± 1.4	1.4 ± 1.0	0.170
Q2. Nocturia	2.1 ± 0.5	1.4 ± 0.7	< 0.001	2.1 ± 0.6	2.2 ± 0.6	0.103
Q3. Urgency	2.3 ± 0.5	2.0 ± 0.7	< 0.001	2.2 ± 0.4	2.3 ± 0.5	0.161
Q4. Urgency incontinence	1.3 ± 1.0	1.1 ± 1.0	0.003	1.3 ± 0.8	1.3 ± 0.8	0.327
Total score	6.9 ± 1.0	5.1 ± 2.2	< 0.001	6.8 ± 2.0	7.2 ± 1.8	0.020

*R group* restricted group, *N-R group* non-restricted group, *SUNa* Na concentration in the spot voiding urine, *Est. 24-h U-Na* estimated 24-h urinary Na, *UV* urine volume, *OABSS* overactive bladder symptom score, *QI, Q2, Q3, and Q4* questions 1, 2, 3, and 4, respectively.

After the 12-week nutritional guidance, the questionnaire scores for the OAB symptoms and the total OABSS improved in the R group (Table 2). However, the questionnaire scores for the OAB symptoms did not change and the total OABSS increased after 12 weeks in the N-R group. Of the 98 patients included, 26 (26.5%) experienced an improvement in the OABSS for urgency by 1 point or more. Furthermore, the urgency score decreased in 24 patients (33.8%) and 2 patients (7.4%) in the R and N-R groups, respectively; this difference in the reduction was statistically significant (P = 0.001). After the 12-week study period, 17 patients (23.9%) in the R group no longer met the criteria used to define OAB in this study, while none of the patients in the N-R group deviated from these criteria.

## Discussion

The present study’s findings showed that reducing the salt intake effectively improved the subjective and objective symptoms in patients with OAB whose daily salt intake was excessive. In addition, while some patients in the R group no longer met the criteria for OAB diagnosis after the 12-week study, none of the OAB symptoms improved in the N-R group. Many researchers have reported that MetS and lifestyle-related diseases are involved in the development of LUTS, including OAB^6,7,12^. In addition, typical lifestyle-related diseases, for example, hypertension, diabetes, and dyslipidemia, are strongly associated with an excessive daily salt intake^10,11^. Indeed, in a previous study, we reported that an excessive daily salt intake was closely associated with LUTS, including nocturia^18^. We found a significantly positive association between nocturnal polyuria and the salt intake and lower voided volumes in subjects with excessive salt intakes as opposed to in those with low salt intakes^18^. In addition, we also found that the OAB symptom scores for the daytime frequency, nighttime frequency, urinary urgency, and urge incontinence (which were determined using the Core Lower Urinary Tract Symptom Score questionnaire^19^) were significantly worse in a group of patients with high salt intakes as compared to in a group of patients with low salt intakes^18^. Another prospective study showed that reducing the salt intake might improve nocturia and the OAB symptoms^12^. Hence, we speculated that there may be a relationship between OAB and excessive salt intakes. However, the aforementioned studies did not specifically focus on patients with OAB, and thus, we designed this study to evaluate the efficacy of restricting the salt intake in patients with OAB whose salt intakes were excessive.

The mechanism underlying the development of OAB due to excessive salt intake remains unclear. However, certain studies have provided some insight into it. In one study, the voided volume of spontaneously hypertensive and salt-sensitive rat models with an excessive salt intake decreased, while their urinary frequency increased after receiving a hyper load of salt^13,14^. Furthermore, Kurokawa et al. reported that the symptoms associated with urine storage may be induced by increased levels of adenosine triphosphate (ATP) and prostaglandin E_2_ (PGE_2_) that are caused by the suppression of the bladder’s blood flow in salt-sensitive rats fed with a high-salt diet^14^. They also reported that the increase in the release of ATP and PGE_2_ from the urothelium due to bladder stretching is likely to play a crucial role in storage dysfunction via stimulation of the bladder afferent c-fibers. In addition, several investigators have reported that excessive salt intake is associated with an imbalance between the sympathetic and parasympathetic activities, which affects the urinary condition^20,21^.

Excessive oxidative stress is associated with OAB symptoms. The relationship between oxidative stress and excessive salt intakes has been thoroughly researched in animal models, and it is believed that excessive oxidative stress, which could be caused by a high salt intake, may affect the OAB symptoms^22,23^. Briefly, in vivo and in vitro studies’ have shown that oxidative stress alters the physiological function of the urothelium and increases bladder sensitivity or the sense of urgency and that the high salt intake is associated with excessive oxidative stress^24^. Moreover, findings from basic research have shown that a high salt loading induces urinary storage dysfunction by upregulating the epithelial Na channel in the bladder epithelium of Dahl salt-sensitive rats^13^. Thus, we believe that complex mechanisms regulate urinary urgency and urinary incontinence secondary to excessive salt intakes.

Many patients with MetS and lifestyle-related diseases that are closely associated with OAB, including hypertension and diabetes, are salt sensitive^25–27^. Interestingly, some normotensive individuals are considered salt-sensitive, and their blood pressures increase after a high salt intake^28^.

Salt sensitivity may be enhanced by the presence of angiotensinogen M235T (T allele), α-adducin Gly460Trp (Trp allele), aldosterone synthase C-344T (T allele), or G protein β3 C825T (T allele) polymorphisms^29^. The Japanese tend to be more salt sensitive as compared to the other ethnic groups^29^. Hence, many patients in our study may have been salt sensitive, and their OAB symptoms may have disappeared when their blood pressure and blood glucose levels stabilized. Unfortunately, there are no tools available for use in a clinical setting that can predict the presence or absence of salt sensitivity in humans.

A previous prospective study showed that while excessive fluid intakes exacerbated the symptoms associated with OAB, restricting the fluid intake alleviated these symptoms and reduced the frequency of detrusor overactivity^7,30^. In addition, in our current study, it is possible that the OAB symptoms were relieved in patients who succeeded in reducing their salt intake due to a decrease in the water and salt intakes. As noted above, various mechanisms may improve OAB symptoms secondary to excessive salt intake. In fact, in the R group, the voided volume increased by approximately 12.6 mL, which was a statistically significant improvement; however, the magnitude of a clinically meaningful improvement in terms of patient satisfaction remains unclear. Previous large-scale clinical trials have reported that the administration of β3-adrenergic receptor agonists and anticholinergic agents to patients with OAB increased the voided volume by 13.07–39.52 mL^31,32^. Hence, the efficacy of salt reduction in terms of increasing the voided volume may be slightly weaker than the efficacy of medication therapy. Furthermore, even in the R group, the OABSS for urinary urgency (an essential symptom of OAB) was only improved by approximately 0.3 points. In this study, 67.6% of the patients in the R-group were on medications for OAB; 14 of these (29.2%) experienced an improvement in urgency as opposed to the 10 patients (43.5%) who were not on medication (P = 0.023). As a result, after the 12-week study period, only 23.9% of the patients who successfully reduced their salt intake did not meet the criteria used to diagnose OAB at the beginning of the study. Hence, a single behavioral therapeutic intervention and medications, especially for refractory symptoms, are not sufficient for treating OAB; OAB should also be managed using comprehensive treatment programs.

There were some limitations to our study. First, the number of participants was relatively small, and we did not include a placebo control group. However, this is the first study to evaluate the efficacy of salt intake reduction in the management of patients with OAB. Furthermore, it would be impossible to conduct a randomized, placebo-controlled study on salt intake levels due to taste concerns and the effects on human health. In addition, we did not investigate, in detail, the effects of successful salt reduction on systematic comorbidities (including the blood pressure and BMI), oxidative stress, and neurotransmitters. Changes in these parameters, secondary to salt restriction, might influence the improvement of OAB. As a future direction, evaluation of the changes in the oxidative stress, blood pressure, and hormones due to salt intake reduction is important to validate our results. Moreover, we will have to investigate the ongoing effects of restricting the salt intake by studying larger populations of patients for longer periods, such as 3 years or more. In addition, we must also investigate the influence of other internal OAB-associated factors, such as the gut microbiome and serum serotonin levels^33,34^, on the salt-dependent urinary function.

Finally, it is important to emphasize that reducing the salt intake for managing OAB is a pharmaceutical-free, safer, and more economic approach as compared to medication and surgery. This approach would minimize the risk to health in most individuals; it is beneficial for many different diseases and could improve survival and the quality of life. Hence, we believe that salt intake reduction should be recommended for patients with OAB in routine medical practice.

## Conclusion

Our study showed that restricting the salt intake can improve urinary symptoms and the overall health of patients with OAB who have an excessive salt intake. Although the biological mechanisms underlying these findings are not fully understood, we suggest that salt intake reduction be recommended before conventional treatments are administered to these patients, because it is a safe and beneficial intervention for human health, and rules out the costs associated with pharmaceutical treatments.

## Data Availability

Data is available for revision.
